# Temporal Organization of Sound Information in Auditory Memory

**DOI:** 10.3389/fpsyg.2017.00999

**Published:** 2017-06-19

**Authors:** Kun Song, Huan Luo

**Affiliations:** ^1^Department of Connectomics, Max Planck Institute for Brain ResearchFrankfurt, Germany; ^2^School of Psychological and Cognitive Sciences, Peking UniversityBeijing, China; ^3^IDG/McGovern Institute for Brain Research, Peking UniversityBeijing, China; ^4^Beijing Key Laboratory of Behavior and Mental Health, Peking UniversityBeijing, China

**Keywords:** temporal scale, temporal organization, auditory memory, unsupervised noise memory paradigm, memory transferring

## Abstract

Memory is a constructive and organizational process. Instead of being stored with all the fine details, external information is reorganized and structured at certain spatiotemporal scales. It is well acknowledged that time plays a central role in audition by segmenting sound inputs into temporal chunks of appropriate length. However, it remains largely unknown whether critical temporal structures exist to mediate sound representation in auditory memory. To address the issue, here we designed an auditory memory transferring study, by combining a previously developed unsupervised white noise memory paradigm with a reversed sound manipulation method. Specifically, we systematically measured the memory transferring from a random white noise sound to its locally temporal reversed version on various temporal scales in seven experiments. We demonstrate a U-shape memory-transferring pattern with the minimum value around temporal scale of 200 ms. Furthermore, neither auditory perceptual similarity nor physical similarity as a function of the manipulating temporal scale can account for the memory-transferring results. Our results suggest that sounds are not stored with all the fine spectrotemporal details but are organized and structured at discrete temporal chunks in long-term auditory memory representation.

## Introduction

Memory is a constructive and organizational process (Schacter et al., 1998; Schacter and Addis, 2007a,b). Taking vision as an example, it is known that instead of being stored with every detail, spatial information is represented in terms of gross spatial structures (Moser et al., 2008). Although visual memory is sensitive to details that are related with semantic processing (Brady et al., 2008); the spatial information is not stored pixel by pixel in visual memory, but gets organized in reference to certain spatial scales (Kjelstrup et al., 2008; Derdikman and Moser, 2010; Rowland and Moser, 2014). In contrast to vision, it is well acknowledged that auditory sounds are characterized by rich temporal dynamics, and time dimension plays a central role in organizing auditory inputs into temporal chunks of appropriate length. It is therefore natural to propose that there might exists critical temporal scales that structure auditory stimuli in memory.

Sussman and Gumenyuk (2005) used a non-speech auditory sequence to study listener’s temporal organization. They found that the auditory MMN cannot be elicited when the inter-stimulus-interval is larger than 200 ms (Sussman and Gumenyuk, 2005; Van Zuijen et al., 2005), suggesting that 200 ms is important for listener’s auditory organization. However, stimuli employed in these studies contain explicit temporal structures and it is difficult to assess whether the observed characteristic temporal scale derives from the dynamic properties of the stimuli themselves or they indeed represent general and intrinsic properties of auditory memory. Interestingly, several studies have shown that human subjects could form long-lasting memories even for random white noise sound, which contains neither semantic labels nor prominent acoustic features and it would be suitable to investigate auditory memory without interfering from semantic processing (Guttman and Julesz, 1963; Hanna, 1984; Warren et al., 2001; Kaernbach, 2004; Goossens et al., 2008, 2009; Agus et al., 2010; Agus and Pressnitzer, 2013; Kumar et al., 2014; Viswanathan et al., 2016). Specifically, one randomly generated white noise sound, as a ‘frozen noise,’ implicitly reoccurred among other non-reoccurring white noises. After repeated exposure, behavioral performance for the ‘frozen noise’ was increasingly enhanced compared to other non-reoccurring noises, suggesting a gradual formation of auditory memory for the re-occurred white noise (Agus et al., 2010). Moreover, the formed memory can last for several weeks indicating its consolidation into long-term memory. Most interestingly, recognition of the memorized noise can tolerate distortions in both spectral and temporal dimension to some extent, implicating that the stored memory for the specific white noise is not represented with all the spectrotemporal details, but instead gets organized at certain resolutions, as previously hypothesized.

The neural correlates of the white noise learning have also been examined using MEG or EEG on human subjects recently (Keceli et al., 2012; Luo et al., 2013; Andrillon et al., 2015). By employing the same experimental paradigm and white noise stimuli, a recent MEG experiment demonstrates that the establishment of a reliable neuronal phase pattern in low-frequency (3–8 Hz) auditory cortical responses mirrors the noise memory formation process. Specifically, with repeated exposure, original novel white noises are memorized and gradually produce robust phase responses in auditory cortex. Given that the neuronal oscillatory phase is known to reflect cyclic cortical excitability states and acts as a temporal reference frame to organize continuous inputs into units, the results thus suggest that the brain forms more reliable temporal segmentation pattern for the memorized white noise (Luo et al., 2013). Linking to previous temporal scale hypothesis, the results also suggest that the brain may gradually establish robust temporal organization for the memorized auditory inputs at certain temporal scales.

In the present study, we designed a series of behavioral experiments to examine the associated temporal scales in long-term auditory memory, by combining previous unsupervised noise memory paradigm (Agus et al., 2010; Luo et al., 2013; Andrillon et al., 2015) with the reversed sound manipulation method (Saberi and Perrott, 1999). In each trial, listeners were asked to determine the noise type (RN sound or N sound). Critically, one particular RN sound (i.e., sound A) was randomly chosen and implicitly presented repeatedly among other noise sounds that were played only once. Previous work has shown that auditory memory for the particular noise could be gradually formed after repeated exposures (Agus et al., 2010).

Here, each experimental block consisted of two concatenated experimental phases without break between them: learning phase and testing phase. During the learning phase, one particular noise (e.g., sound A) was presented repeatedly among others and subjects presumably would form implicit memory for the sound. Next, during the subsequent testing phase, the memorized noise A was replaced by noise A′, which was generated from sound A by a reversed sound manipulation method (Saberi and Perrott, 1999). Specifically, original sound A was first divided into chunks of certain temporal scale, and the sound signals within each chunk was then locally reversed in time. Next we systematically measured the A-to-A′ memory transferring performance at different temporal scales (e.g., from 31.25 to 500 ms).

If auditory memory is mediated at certain temporal scale and sound A′ is modified from sound A just at this critical temporal scale, the reversed manipulation would largely disrupt temporal representation of sound A in auditory memory and sound A′ would be treated as a completely new sound. As a result, the formed memory for sound A during the learning phase would not transfer to sound A′ during the testing phase. On the other hand, when the temporal scale employed in the modification is far away from the critical scale in auditory memory, sound A′ would maintain critical information about sound A in memory representation, and the formed memory for sound A would in turn be able to transfer to sound A′ during the testing phase. In other words, although sound A′ and sound A are acoustically distinct, they are similar in memory representation and are thus interchangeable in memory performance. This phenomenon was investigated previously and the authors concluded that the memory for a 500 ms noise segment can be transferred to its total reversal form (Agus et al., 2010). However, in previous work, the authors only showed that A′A′ showed memory effect; it is not clear whether this effect is a reflection of memory transfer or re-learning of the temporal manipulated sound. We added a new refRN sound BB in the testing phase; the BB sound would be re-learned after repeated exposure. We thus can investigate the AA to A′A′ memory transfer by comparing the listeners’ performance on A′A′ and BB. In summary, if auditory memory is mediated by certain temporal scale, we would expect to see a U-shape A-to-A′ memory transferring performance. In contrast, if there does not exist specific memory-associated temporal scale, a monotonically decrease in A-to-A′ memory transferring performance with increase in temporal scale in sound manipulation would be expected.

## Materials and Methods

### Participants

One hundred and forty-seven adults aged 18–24 participated in the memory transform experiments (Experiment 1–7). Sixteen subjects participated in Experiment 1. Fifteen subjects participated in Experiment 2. Fifteen subjects participated in Experiment 3. Sixteen subjects participated in Experiment 4. Sixteen subjects participated in Experiment 5. Twenty-two subjects participated in Experiment 6. Seventeen subjects participated in Experiment 7. Twenty-six subjects participated in the perceptual discrimination experiments (Experiment 8–9). All participants had normal hearing and had no history of psychiatric or neurological disorders. All participants provided the informed consent form, approved by the Research Ethics Committee at Institute of Biophysics, Chinese Academy of Sciences.

### Sound Stimuli

For all the experiments, the sound stimuli were generated using MATLAB R2009a (MATHWORKS^®^), with the sampling frequency of 44.1 kHz, and were presented at a comfortable sound level. Experiments were performed and controlled by the Psychtoolbox 3.0 toolbox (Brainard, 1997; Pelli, 1997). Sound stimuli were presented through Sennheiser HD215 headphone on a Dell OPTIPLEX380 PC. All the participants performed the experiments in an acoustically shielded sound-proof room.

#### Memory Transferring Experiments (Experiment 1 ∼ Experiment 7)

**FIGURE 1:**
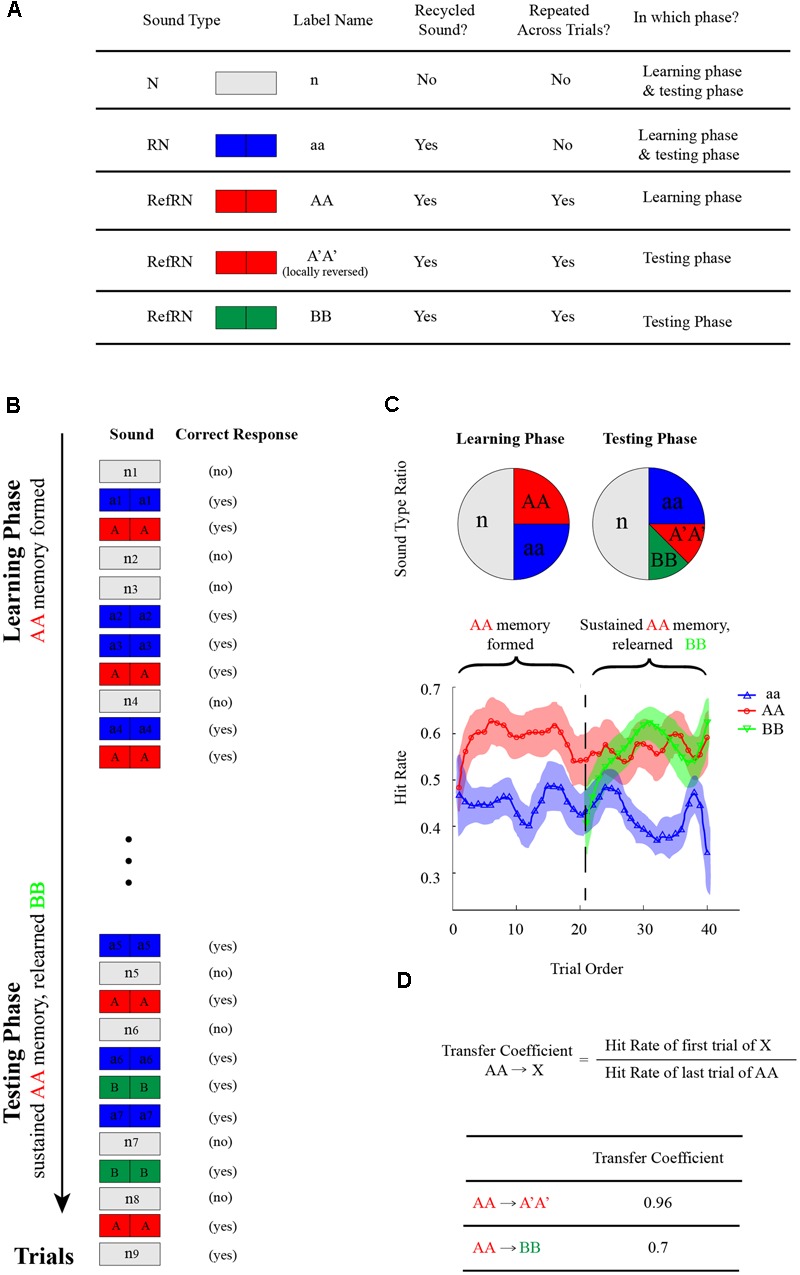
Memory-transferring experimental paradigm (Experiment 1–7). **(A)** Stimulus types in the memory-transferring experiments. N-type (n, white rectangle): a 1 s running noise; RN-type (aa, blue divided rectangle): a noise sample generated by concatenating two identical 0.5 s noise segments. There were four classes of RN-type sound: RN (aa, blue divided rectangle): generated anew in each trial and presented only once; RefRN (AA, red divided rectangle): one particular exemplar of the RN sounds that reoccurred across trials during the learning phase; RefRN (A′A′, red divided rectangle): a modified version of original AA sound by the reversed sound manipulation, which reoccurred during the testing phase; RefRN (BB, green divided rectangle): another particular exemplar of the RN sounds that reoccurred across trials during the testing phase but newly generated independent of previous AA sound. **(B)** In each trial throughout each experimental block, subjects were either presented with a RN-type sound or an N-type sound and were asked to determine the noise type (RN or N) by pressing corresponding keys (Yes or No). Critically, one particular exemplar of the RN sounds reoccurred, interspersed throughout each experimental block (RefRN, red divided rectangle; AA sound). During the subsequent testing phase, A′A′ sound (red divided rectangle), which was also a RefRN type sound and was a modified version of AA sound by the reversed sound manipulation method, replaced the original AA sound and reoccurred throughout each experimental block. In addition, as a control, a BB sound (green divided rectangle), which was also a RefRN type sound but was newly generated independent of original AA sound, also reoccurred throughout each block. **(C)** Upper: the ratio of N:RN:RefRN was set as 2:1:1 to keep the two noise types balanced (same Yes and No correct response) during the learning phase; the ratio of N:RN:A′A′:BB was set as 2:1:0.5:0.5 to keep the two noise types balanced (same Yes or No correct response) during the testing phase. Lower: an example of hitting performance as a function of trial order throughout experiment block (blue line: aa; red line: AA during the learning phase and A′A′ during the testing phase; green line: BB during the testing phase). Note the gradually formed memory for AA during the learning phase (the red plots, trial 1–20). Critically, during the testing phase (trial 21–40), the formed memory for AA successfully transferred to A′A′ (the red plots closed to the vertical line around trial 20), whereas the BB showed a re-learning process (green line, gradually rising performance). **(D)** Definition of memory-transferring coefficient: the hit rate ratio between the early trials of A′A′ (or BB) during testing phase and the late trials of AA during learning phase.

As illustrated in **Figures 1A,B**, listeners were either presented with a noise sample generated by concatenating two identical 0.5 s noise segments (RN, blue divided rectangle) or a 1 s running noise (N sound, white rectangle) and were asked to determine the noise type (RN or N) by pressing corresponding keys (Yes or No). Critically, one particular exemplar of the RN sounds reoccurred, interspersed throughout each experimental block (RefRN, red divided rectangle; AA sound). As shown in the left panel of **Figure 1C**, during the learning phase, the ratio of N:RN:RefRN was set as 2:1:1 to keep the two noise types balanced (same Yes and No correct response). During the subsequent testing phase (right panel of **Figure 1C**), A′A′ sound (red divided rectangle), which was also a RefRN type sound and was a modified version of AA sound by the reversed sound manipulation method, replaced the original AA sound and reoccurred throughout each experimental block. In addition, as a control, a BB sound (green divided rectangle), which was also a RefRN type sound but was newly generated independent of original AA sound, also reoccurred in testing phase throughout each block. The ratio of N:RN:A′A′:BB was set as 2:1:0.5:0.5 to keep the two noise types balanced (same Yes or No correct response).

**FIGURE 2:**
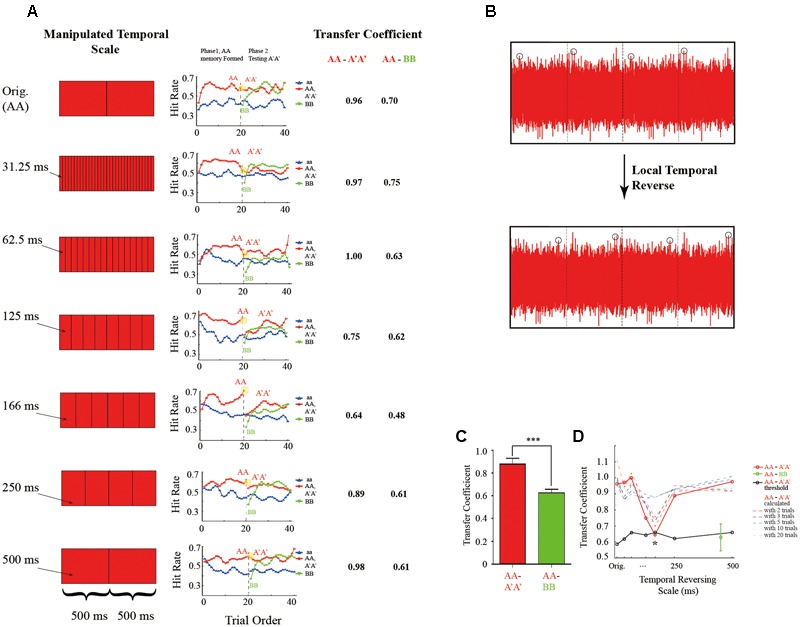
Memory-transferring performance at various reversed temporal scales. **(A)** Left: illustration of AA-to-A′A′ manipulation at various temporal scales (from top to bottom: Original, 31.25, 62.5, 125, 166 ms, and 250–500 ms). Specifically, sound AA was first divided into chunks of certain temporal scale, and the sound signals within each chunk was then locally reversed in time **(B)**. Middle: grand averaging hitting performance as a function of trial order throughout experiment block (blue line: aa; red line: AA during the learning phase and A′A′ during the testing phase; green line: BB during the testing phase) at different manipulating temporal scales (from top to bottom: Original, 31.25, 62.5, 125, 166 ms, and 250–500 ms, corresponding to Experiment 1–7, respectively). Note the gradually formed memory for AA during the learning phase (the red plots, trial 1–20) under all conditions. Critically, during the testing phase (trial 21–40), the BB showed a re-learning process (green line, gradually rising performance) for all the conditions. However, the AA-to-A′A′ memory transferring performance differed across different manipulating temporal scales (the red plots closed to the vertical line around trial 20). Note a large decrease in A′A′ performance for temporal scales closed to 125 and 166 ms. Right: the calculated AA-to-A′A′ and AA-to-BB memory transferring coefficient for different manipulating temporal scales. **(B)** Illustration of local temporal reversed method. **(C)** Bar plots of AA-to-A′A′ (red bar) and AA-to-BB (green bar) memory-transferring coefficient averaged across different manipulating temporal scales. **(D)** Grand averaged AA-to-A′A′ (red line) and AA-to-BB (blue line) memory transferring coefficient as a function of manipulating temporal scales. Note the U-shape pattern for AA-to-A′A′ memory transferring performance with the minimum value around 166 ms and the flat pattern for AA-to-BB memory transferring performance. The asterisks denote statistically significance (*p* ≤ 0.05, permutation test).

Seven experiments (Experiment 1–7) were run on different subject groups, and each experiment consists of four experimental blocks. Those experiments mainly differ in the temporal scale employed in the reversed sound manipulation method to generate A′A′ from AA, except in Experiment 1 the A′A′ was exactly the AA (Orig condition). Specifically, as shown in **Figures 2A,B**, AA was first divided into chunks of certain temporal scale, and the sound signals within each segment was then locally flipped in time to generated A′A sound. The temporal scale used in Experiment 2 to Experiment 7 was 31.25, 62.5, 125, 166.6, 250, and 500 ms, respectively. The tasks and the stimulus ratio was the same in all the experiments (Experiment 1 to Experiment 7, left panel of **Figure 2A**).

#### Perceptual Similarity Experiment (Experiment 8 ∼ Experiment 9)

Different from memory transferring experiment, all the sound stimuli in the perceptual similarity experiments were generated afresh in each trial and were only presented once, and there were no RefRN-type sounds.

### Experimental Procedures

Here we combined previous unsupervised noise memory paradigm (Agus et al., 2010) with the reversed sound manipulation method (Saberi and Perrott, 1999) to examine the associated temporal scales in auditory memory.

#### Unsupervised Noise Memory Paradigm

As illustrated in **Figures 1A,B**, listeners were presented with either a RN-type sound (blue divided rectangle) or a N-type sound (white rectangle) and were asked to determine the noise type (RN or N) by pressing corresponding keys (Yes or No). Critically, one particular exemplar of the RN sounds reoccurred, interspersed throughout each experimental block (RefRN, red divided rectangle; AA sound), whereas other RN-type sounds were generated afresh in each trial and presented only once. Previous studies have shown that auditory memory could be formed implicitly for the reoccurring sound (i.e., AA sound here) after repeated exposure.

#### Memory Transferring Experiments

Seven experiments (Experiment 1–7) were run on different subject groups, and each experiment consists of 4 experimental blocks, each of which contained 160 trials. As illustrated in **Figure 1**, each experimental block consisted of a learning phase and a testing phase, which were concatenated with each other and contained no breaks between them (**Figure 1B**). There were in total 80 trials (40 trials of different N sounds, 20 trials of different RN sounds, and 20 trials of one particular AA sound) in the learning phase. As demonstrated in previous studies, subjects could form auditory memory for RefRN sound (i.e., AA sound) to some extent (Left panel of **Figure 1C**).

The testing phase started right after the learning phase (**Figure 1B**). There were in total 160 trials in the leaning phase (80 trials of different N sounds, 40 trials of different RN sounds, 20 trials of one particular AA sound, 20 trials of one particular BB sound). Note that the A′A′ sound here was the temporally manipulated version of AA sound and was used as a probe to assess the memory-transferring performance from the learning phase to testing phase (i.e., from AA to A′A′). In addition, to disambiguate possible newly formed memory effects (repeated presentation of A′A′) on the AA-to-A′A′ memory-transferring performance, another RefRN sound (i.e., the BB sound) that was generated anew and was independent of AA sound, was presented during the testing phase. In other words, the learning phase contained only one RefRN-type sound (sound AA), whereas in the testing phase, there were two RefRN-type sound (sound A′A′ and sound BB), both of which occurred repeatedly across trials but differed in their relationship to the original AA sound. Specifically, A′A′ was the locally reversed version of AA sound and BB was independent of AA sound.

#### Perceptual Similarity Experiments

Two experiments (Experiment 8–9) were run on different subject groups, and each experiment consisted of 4 experimental blocks, each of which contained 160 trials.

**FIGURE 3:**
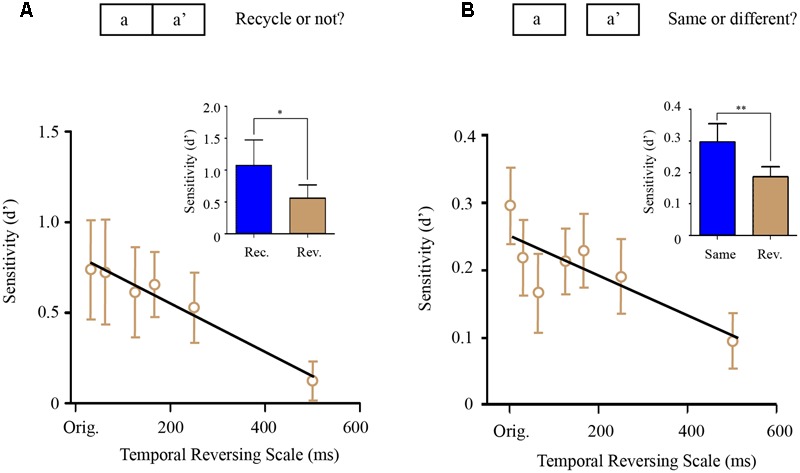
Perceptual similarity experiments (Experiment 8–9). **(A)** Segment-repetition detection experiment (Experiment 8). There were three types of sound stimulus (1/3 N-type sounds, 1/3 RN-type sounds, and 1/3 mixed-type sounds). Subjects were asked to determine whether the sound contained two repetitive segments or not. Each sound was generated anew in each trial and presented only once in the experiment. Upper: the mixed-type sound (i.e., aa′) was generated by seamlessly concatenating one 500 ms noise segment **(A)** and its locally temporally manipulated version (a′). Lower: grand averaged repetition detection sensitivity for the mixed-type sounds (aa′) as a function of temporally reversed scale with a linear fitting line. Inserted figure: detection sensitivity of the RN-type sound (blue bar, rec.) was significantly larger than that of the mixed-type sounds (gray bar, rev.). **(B)** Same-different judging experiment (Experiment 9). Upper: subjects were presented with two 500 ms noise segments with 1 s interval between them, and were asked to determine whether the two noise segments were the same or different. The two noise segments were the same (same-sound trial), or totally different (different-sound trial), or a noise segment **(A)** and its temporally manipulated version (a′) respectively (the mixed-type trial). Lower: grand averaged same-different detection sensitivity for the mixed-type trials (a and a′) as a function of temporally reversed scale with a linear fitting line. Inserted figure: detection sensitivity of the same-sound trials (blue bar, rec.) was significantly larger than that of the mixed-type trials (gray bar, rev.). The asterisks ^∗^ denotes statistically significance (*p* < 0.05), ^∗∗^ denotes statistically significance (*p* < 0.01).

Repetition-detection experiment (Experiment 8) consisted of three experimental blocks, each of which contained 360 trials (120 N-type sounds, 120 RN-type sounds, and 120 mixed-type sounds). The 360 sound stimuli were generated anew in each trial and was presented only once in the experiment. The N-type sound and RN-type sounds were the same as that in memory transferring experiments such that N-type sound was a 1 s running noise (the correct answer would be No) and the RN-type sound was a sound consisted of two same 500 ms noise segments (the correct answer would be Yes). Notably, the mixed-type sound (i.e., aa′) was generated by seamlessly concatenating one 500 ms noise segment (a) and its locally temporally manipulated version (a′). Subjects were asked to determine whether the sound they heard on each trial contained two repetitive segments or not and the existence of the mix-type-sound (aa′) were unbeknownst to them (aa′) (**Figure 3A**). The logic here is that the more the a′ segment was perceived to be similar to the a segment, the more the subjects would respond with yes. Specifically, for the mixed-type sounds, there were six possible reversed temporal scales (i.e., 31.25, 62.5, 125, 166, 250, and 500 ms) employed to convert segment a into segment a′.

The same-different judging experiment (Experiment 9) consisted of 4 experimental blocks, each of which contained 360 trials. In each trial, participants were presented with two 500 ms noise segments with 1 s interval between them, and were asked to determine whether the two noise segments were the same or different. In one third of trials, the two noise segments were the same (the correct answer would be Yes), and in another one third of trials, the two noise segment were different (the correct answer would be No). Importantly, there were also one third of mixed-type trials (**Figure 3B**), in which the two noise segments presented in one trial were a noise segment (a) and its temporally manipulated version (a′). The logic here is that the more the a′ segment was perceived to be similar to the a segment, the more subjects would perceive that they were same. Specifically, for the mixed-type trials, there were 6 possible reversed temporal scales (i.e., 31.25, 62.5, 125, 166, 250, and 500 ms) employed to convert segment a into segment a′.

### Data Analysis

#### Memory-Transferring Coefficients

In order to visualize the learning process, we calculated the grand averaged hit rates of aa, AA (in learning phase), and A′A′, BB (in testing phase) on each trial. Listeners’ response for sound aa, AA, A′A′, and BB was first averaged across the four blocks, respectively, to get a hit rate curve for each sound (Agus et al., 2010); and the hit rates for every sound were then averaged across listeners. Notably, given that any RefRN sound could be relearned after repeated exposure, hitting performance during the early trials rather than late trial in the testing phase would be most sensitive to tell whether the A′A′ performance derives from previously formed memory or relearning process.

The memory-transferring coefficient was calculated using the ground average hit as:

Transfer Coefficient AA→X=Hit rate of first trial of XHit Rate of last trial of AA

Here, X could be A′A′ or BB. Hit rate calculation was corrected by replacing extreme value 0, 1 with 1/(2^∗^n) and 1-1/(2^∗^n) respectively, where n was the number of trials (Macmillan and Creelman, 2005). Note that the memory-transferring coefficients were also calculated based on other performance parameters (e.g., d prime, number of trials included in the calculation) and similar results were obtained (**Supplementary Figure S2**).

A permutation test was performed to examine which manipulating temporal scale significantly disrupted memory-transferring performance. Specifically, the raw data was randomly reshuffled across temporal scale conditions, and the memory-transferring coefficients were recalculated. This permutation was done 200 times, resulting in a distribution of memory-transferring coefficient for each temporally reversed scale. Based on the 200-point distribution of memory-transferring coefficient for each temporal scale, the 0.05 threshold was set to test the statistically significance of the original memory-transferring coefficient.

#### Perceptual Similarity Experiments

For the repetition-detection experiment (Experiment 8), we calculated the detection sensitivity (d′) (Macmillan and Creelman, 2005) of the RN-type sounds relative to N-type sounds, as well as the d′ of the mixed-type sounds relative to N-type sounds at each temporal manipulation scale. Here the hit rates were calculated as the ratio between the number of response “yes” in the total number of RN-type or mixed-type sounds. And the false rate was calculated as the ratio the response “yes” in the total number of N-type sounds (Agus et al., 2010). For the same-different judging experiment (Experiment 9), we calculated the detection sensitivity d′ of the same-sound trials relative to the different-sound trials, as well as the mixed-type trials relative to the different-sound trials, at each temporal manipulation scale, respectively. The hit rate and false alarm rate were similarly calculated as in experiment 8.

## Results

### Memory Maintaining and Sound Relearning (Experiment 1)

**Figure 2A** plots the grand-averaged hitting performance as a function of trial order (trial 1 to trial 40) throughout each experiment block in the memory transferring experiments (Experiment 1–7). As shown in the middle panel of **Figure 2A**, each experimental block consists of a learning phase (first 20 trials) and a testing phase (last 20 trials). Note that at the start of each block, the RN sound (i.e., aa sound, blue line) and the RefRN sound (i.e., AA sound, red line) did not differ much in hitting performance. However, after several trials of repeated exposure to the particular AA sound in the learning phase (trial 1–20), the hitting performance for the AA sound (red line) began to develop increasingly compared to the aa sound (blue line), suggesting that the AA sound had begun to be learned and stored into auditory memory, consistent with previous findings (Agus et al., 2010; Luo et al., 2013).

Next and critically, during the testing phase right after the learning phase (trial 21–40), two RefRN sounds were presented (A′A′, red line; BB, green line) in addition to the RN sounds (aa sound, blue line). Both of A′A′ and BB reoccurred throughout experimental block but they differed in their relationship to the original presented AA sound in the testing phase. Specifically here in Experiment 1 (top panel of **Figure 2A**), sound A′A′ was exactly the same as the original AA sound, whereas sound BB was generated independently of the sound AA. As shown in the top panel of **Figure 2A**, it is quite clear that the formed memory for AA during the learning phase was successfully maintained on sound A′A′ (red line) at the start of the testing phase (starting from trial 21). The results were reasonable because here in Experiment 1, sound A′A′ was the same as sound AA. In contrast, the hitting performance for sound BB (green line) started from the same level as that of the RN sound (blue line), and gradually increased after repeated exposure, thus supporting a re-learning process for the new BB sound. In summary, both sound A′A′ and BB during the testing phase showed better hitting performance than RN sounds, indicating memory formation for both of them. However, they were mediated by different processes, i.e., A′A′ memory was actually transferred from AA memory formed during the learning phase, whereas BB memory was completely re-established during the testing phase.

We then quantified the memory transferring performance from AA (learning phase) to A′A′ or BB (testing phase), by calculating the memory transfer coefficients (**Figure 1D**) for each experiment, respectively (Experiment 1–7). Specifically, the ratio between the hitting performance for A′A′ or BB during the early trials in testing phase (when A′A′ and BB could not be re-learned yet given small repeated trials) and that for AA during the late trials in learning phase (when memory for AA was fully established and stabilized) was calculated (**Figure 1D**). Larger transfer coefficients thus indicate better memory transfer, whereas smaller transfer coefficients represent worse memory transfer. In Experiment 1 when A′A′ was the same as AA, The AA-to-A′A′ memory transfer coefficient was 0.96, larger than that of AA-to-BB (0.7) (**Figures 1D, 2A**).

### Memory-Transferring at Different Reversed Temporal Scales (Experiment 2 to Experiment 7)

We next examined the memory-transferring performance at different manipulating temporal scales (31.25–500 ms, Experiment 2–7) respectively. As illustrated in the middle panel of **Figure 2A**, during the learning phase (the first 20 trials within each experimental block), the memory for AA (red line) was gradually formed compared to the RN sound (i.e., aa sound, blue line) and reached a plateau around the end of the learning phase (close to trial 20). However, right at the start of the testing phase (starting from trial 21), the A′A′ performance varied largely for different manipulating temporal scales (red line). For example, the A′A′ performance kept relatively higher when temporal scale was either small (e.g., 31.25, 62.5 ms) or large (e.g., 250, 500 ms), suggesting that information manipulation on these temporal scales did not efficiently disrupt the previously stored AA representation auditory memory and thus the formed AA memory could successfully transfer to the new A′A′ sounds. In contrast, the A′A′ performance dropped significantly when the manipulating temporal scale was around 166 ms, indicating that information structured at the temporal window is critical in auditory memory. In sum, the AA-to-A′A′ memory transferring performance showed a U-shape curve as a function of reversed manipulating temporal scales with the minimum around 166 ms. In contrast, the BB performance (green plots) in all the experiments (Experiment 1 to 7) all started from a low level similar to the aa performance at the start of the testing phase, suggesting a complete re-learning process.

**Figure 2D** plots the calculated AA-to-A′A′ (red line) and AA-to-BB (green bar) memory transferring coefficients as a function of manipulating temporal scale (31.25–500 ms, Experiment 2–7). As expected, the AA-to-A′A′ showed a U-shape pattern suggesting that the manipulating temporal scales largely influenced the memory transferring such that when sound AA was disrupted at scale of ∼166 ms, the formed memory for AA cannot transfer to A′A′ successfully (red line). On the other hand, the AA-to-BB memory transfer coefficients were at lower level than that of AA-to-A′A′ (**Figure 2C**, *t*(6) = 7.2, *p* < 0.001, **Figure 2D** green bar), further confirming that BB performance was independent of AA performance. Permutation test further confirmed that the memory transfer coefficients at temporal scales of 125 and 165 ms were significantly smaller than the other conditions (**Figure 2D**, *p* ≤ 0.05, two-tailed).

Furthermore, similar U-shape-trend results were obtained when the memory transferring coefficients were calculated based on d′ (**Supplementary Figures S2a,b**). More interestingly, when the memory transfer coefficient was calculated with more trials (e.g., 1–10 trials of A′A′ and the last 1–10 trials of AA), the transfer coefficients at both large and small temporal scales (e.g., 31.25, 500 ms) were almost the same, indicating that listener’s performance on A′A′ kept high from the start of the testing phase to the end. However, the transfer coefficients at temporal scales of 125, 166 ms were increasing when more trials were included in analysis. This indicates that listeners’ performance for those A′A′ became better at the later stage of testing phase. Specifically, when using the total 20 trials of AA′ and 20 trials of AA for calculating transfer coefficient, no significant difference could be found between different temporal manipulating scales, and this is the same result as calculated by d′ and hit rate (**Supplementary Figures S2a,b**). On the contrary, the AA-BB memory transfer coefficient had an increasing trend at all temporal scale when more trials were included for analysis (**Supplementary Figures S2c,d**).

### Perceptual Similarity at Different Reversed Temporal Scales (Experiment 8 to Experiment 9)

The memory transferring experiments (Experiment 1–7) have demonstrated a U-shape memory transferring performance as a function of reversed temporal scale, with the minimum value around 166 ms, suggesting that ∼200 ms (125–250 ms) acts as a fundamental temporal chunk to structure sounds in auditory memory, also consistent with previous findings (Sussman and Gumenyuk, 2005; Van Zuijen et al., 2005). However, the results might also derive from the auditory perceptual similarity between the original AA sound and the manipulated A′A′ sound. For example, sound A′A′, which is the manipulated version of AA sound at 160 ms, might sound maximally distinct from sound AA, compared to other temporal scales, which may lead to the U-shape memory transferring performance. We thus further assessed the perceptual similarity between AA and A′A′ that are manipulated at various reversed temporal scales, in two experiments (Experiment 8–9), to examine whether perceptual dissimilarity performance could account for the U-shape memory performance.

In Experiment 8, we employed a repetition-detection paradigm (**Figure 3A**). Specifically, a random noise segment (sound a) was seamlessly concatenated with itself (a) or with a manipulated version of itself (sound a′, at various reversed temporal scales). Subjects were asked to determine whether the sound stimulus contains two repetitive segments or not. In general, sound aa showed better repetition detection performance than sound aa′ (**Figure 3A**, bar figure, *p* < 0.05). Interestingly, the aa′ repetition detection performance monotonously decreased as the reversed temporal scale increased [**Figure 3A**, line plots, one-way ANOVA, *F*(6,5) = 5.713, *p* < 0.001] and did not show the U-shape pattern as that in the memory transferring experiments (a straight line with negative slope fitted better than a horizontal line, *p* < 0.0107).

In Experiment 9, we employed a same-different judgment paradigm (**Figure 3B**) to assess the perceptual similarity between sound and its temporally reversed version at various reversed temporal scales. Specifically, in each trial, subjects were presented with two sounds -a noise segment (sound a) and its temporally reversed version (sound a′), and were asked to determine whether the two sounds were same or different. As shown in **Figure 3B**, again, instead of a U-shape pattern, the results showed a monotonous decrease as a function of increasing temporal scale (a straight line with a negative slope fitted better than a horizontal line, *p* < 0.0232), similar to the results of Experiment 8.

In summary, the above perceptual similarity experiments (Experiment 8–9) indicates that the perceptual similarity of the noise with its temporal manipulated form decreases as the temporal manipulation scale increases, consistent with previous results (Walker et al., 2008). This indicates that the observed U-shape memory transferring performances (**Figure 2D**) cannot be explained by AA-A′A′ perceptual similarity, but might indeed reflect general temporal scale that structures auditory representation in memory space.

## Discussion

In the present study, we examined the temporal structures of auditory memory by combining a previously developed unsupervised noise memory paradigm with a reversed sound manipulation method (Experiment 1–7). Our results demonstrated a U-shape memory transferring pattern as a function of reversed manipulating temporal scale. Specifically, memory-transferring performance dropped to the minimum level when the manipulating temporal scale was around 200 ms, suggesting that ∼200 ms might be a critical temporal window that organizes auditory information in memory space. The memory-transferring performance remained high when the manipulating temporal scale was deviated away from 200 ms, either smaller or larger. The entire reversal also remained high memory transfer, consistent with previous work (Agus et al., 2010). We further excluded perceptual similarity factors that may contribute to the U-shape memory-transferring results, by performing two control experiments (Experiment 8–9). Our results suggest that sounds are not stored with all the spectrotemporal details but are organized and structured at discrete temporal chunks in auditory memory representation.

### Temporal Structures in Auditory Perception

Auditory sounds are known to be characterized by rich dynamics on multiple temporal scales; meanwhile, it is well acknowledged that auditory perception does not require a detailed analysis of the signal and coarse representation is known to suffice. For example, natural speech recognition can tolerate large extents of distortions (Shannon et al., 1995; Elliott and Theunissen, 2009). Previous MEG studies on human subjects have also revealed that natural speech sounds elicit brain responses with robust neuronal phase patterns in theta band (3–5 Hz), suggesting that the brain segments incoming speech sounds into discrete temporal chunks of ∼200 ms, a temporal scale commensurate with syllable length across languages (Luo et al., 2013). Here we extended previous findings to auditory memory by demonstrating that there also exist critical temporal scales to structure sound information in auditory memory.

### Temporal Structures in Auditory Memory

Previous work has also provided evidence supporting ∼200 ms as a fundamental temporal unit in auditory memory (Sussman and Gumenyuk, 2005; Van Zuijen et al., 2005). However, those findings are possibly due to the dynamic properties or semantic meaning contained in the stimuli. Here we used randomly generated white noise that contains neither semantic label nor explicit temporal structures that may guide memory performance. Our results are thus first consistent with previous work but also add novel evidence supporting the critical role of ∼200 ms in auditory memory.

### Noise Memory and Learning

We employed a unsupervised auditory memory paradigm (Guttman and Julesz, 1963; Hanna, 1984; Kaernbach, 2004; Goossens et al., 2007, 2008; Agus et al., 2010; Agus and Pressnitzer, 2013; Kumar et al., 2014). Interestingly, the quickly formed auditory memory could last for several weeks and tolerate distortions in both spectral and temporal dimension to some extent (Agus et al., 2010; Agus and Pressnitzer, 2013). Our memory-transferring results are thus also in line with those findings, further supporting that the stored long-term memory for the sounds is not represented with all the spectrotemporal details. Meanwhile, different from previous work, our results systematically examined memory performances for sounds that are manipulated at various temporal scales, and extended previous finding by showing that the sounds are organized at temporal chunks of ∼200 ms in length in auditory memory.

One interesting question is what was learned by listeners. Agus and Pressnitzer (2013) suggested that the learning in the noise memory paradigm (Agus et al., 2010) is the 0.5 s noise token itself but not the modulation pattern of the waveform. Studies suggested that the underlying acoustic attribute for the noise memory is confined in a time length from 10 ms (Viswanathan et al., 2016) to about 100 ms (Kaernbach, 1993), suggesting that listeners might depend on some local physical features to form the memory of noise. However, these studies applied specific paradigms such as shuffle noise internally (Viswanathan et al., 2016) or noise feature tapping (Kaernbach, 1993) such that only the local physical features can be studied, and any form of organization of the sound segment will be skipped. Our result suggests a ∼200 ms temporal scale for auditory memory. Combing with evidence from our previous study on the neural mechanism of noise memory (Luo et al., 2013), which suggest a reliable neuronal phase pattern corresponding to a learned noise, we tend to believe that the noise memory also contains a component that represents the sound in an organized form, instead of just as a local feature.

### Neural Mechanism for Auditory Memory

Recently, an MEG study investigating neural mechanisms underlying the noise memory formation showed that the establishment of a reliable neuronal phase pattern in theta-band (3–8 Hz) auditory cortical responses mirrors the noise memory formation process, suggesting that the brain forms a more reliable temporal segmentation pattern for the memorized white noise (Luo et al., 2013). The theta-band corresponds to a temporal window of approximately ∼200 ms, thus also matching well with the present behavioral results. The ∼200 ms scale is also consistent with speech perception (Saberi and Perrott, 1999; van Wassenhove et al., 2007; Remez et al., 2008; Luo and Poeppel, 2012) and music perception (Hickok et al., 2015).

### Physical Similarity Controls

Moreover, our two control experiments (Experiment 8–9) have excluded the perceptual similarity interpretations for the U-shape memory-transferring results. We further quantified the similarity in acoustic property between the noise segment and its temporally reversed version in several ways: sound signals (**Supplementary Figure S1a**), temporal envelopes (**Supplementary Figure S1b**), and spectrum profiles (**Supplementary Figure S1c**). None of these physical similarity values showed a U-shape performance as a function of temporally reversed scales, as that in memory-transferring performance, further supporting that the observed temporal structures in auditory memory are independent of acoustic properties of sound stimuli.

## Ethics Statement

This study was carried out in accordance with the recommendations of the Research Ethics Committee at Institute of Biophysics, Chinese Academy of Sciences with written informed consent from all subjects. All subjects gave written informed consent in accordance with the Declaration of Helsinki. The protocol was approved by the Research Ethics Committee at Institute of Biophysics, Chinese Academy of Sciences.

## Author Contributions

KS took part in experimental design, data collection, data analysis and writing the paper. HL took part in project initiation, experimental design, data analysis, paper writing, and supplying research tools and infrastructure.

## Conflict of Interest Statement

The authors declare that the research was conducted in the absence of any commercial or financial relationships that could be construed as a potential conflict of interest.

## Supplementary Material

FIGURE S1 Physical similarity. **(a)** Left: the Euclidean distances between a noise segment in length of 0.5 s and its local temporal reversing versions at different temporal manipulation scales. Right: the distribution of Euclidean distance between two randomly generated noise segments, calculated from 500 different noise pairs. **(b)** Left: the correlations of the envelope of a noise segment in length of 0.5 s and the envelopes of its local temporal reversing versions at different temporal manipulation scales. Right: the distribution of the correlation between the envelopes of two randomly generated noise segments, calculated from 500 different noise pairs. **(c)** Left: the correlations of the spectrum equivalent bands pattern (ERB pattern) of a noise segment in length of 0.5 s and the envelopes of its local temporal reversing versions at different temporal manipulation scales. Right: the distribution of correlation between the ERB patterns of two randomly generated noise segments, calculated from 500 different noise pairs.

FIGURE S2 Memory-transferring coefficients calculated using other behavioral parameters. **(a)** AA-A′A′ memory-transferring coefficient as a function of temporal reversed scale, calculated using the hit rate averaged across different number of trials. **(b)** AA-A′A′ memory-transferring coefficient as a function of temporal reversed scale, calculated using d′, averaged across different number of trials. **(c)** AA-BB memory-transferring coefficient as a function of temporal reversed scale, calculated using the hit rate averaged across different number of trials. **(d)** AA-BB memory-transferring coefficient as a function of temporal reversed scale, calculated using d′, averaged across different number of trials.
