# Supplementary figures and images for: Temporal Organization of Sound Information in Auditory Memory
Source: Front Psychol. 2017 Jun 19;8:999. doi: 10.3389/fpsyg.2017.00999 (PMC5475238; doi:10.3389/fpsyg.2017.00999)

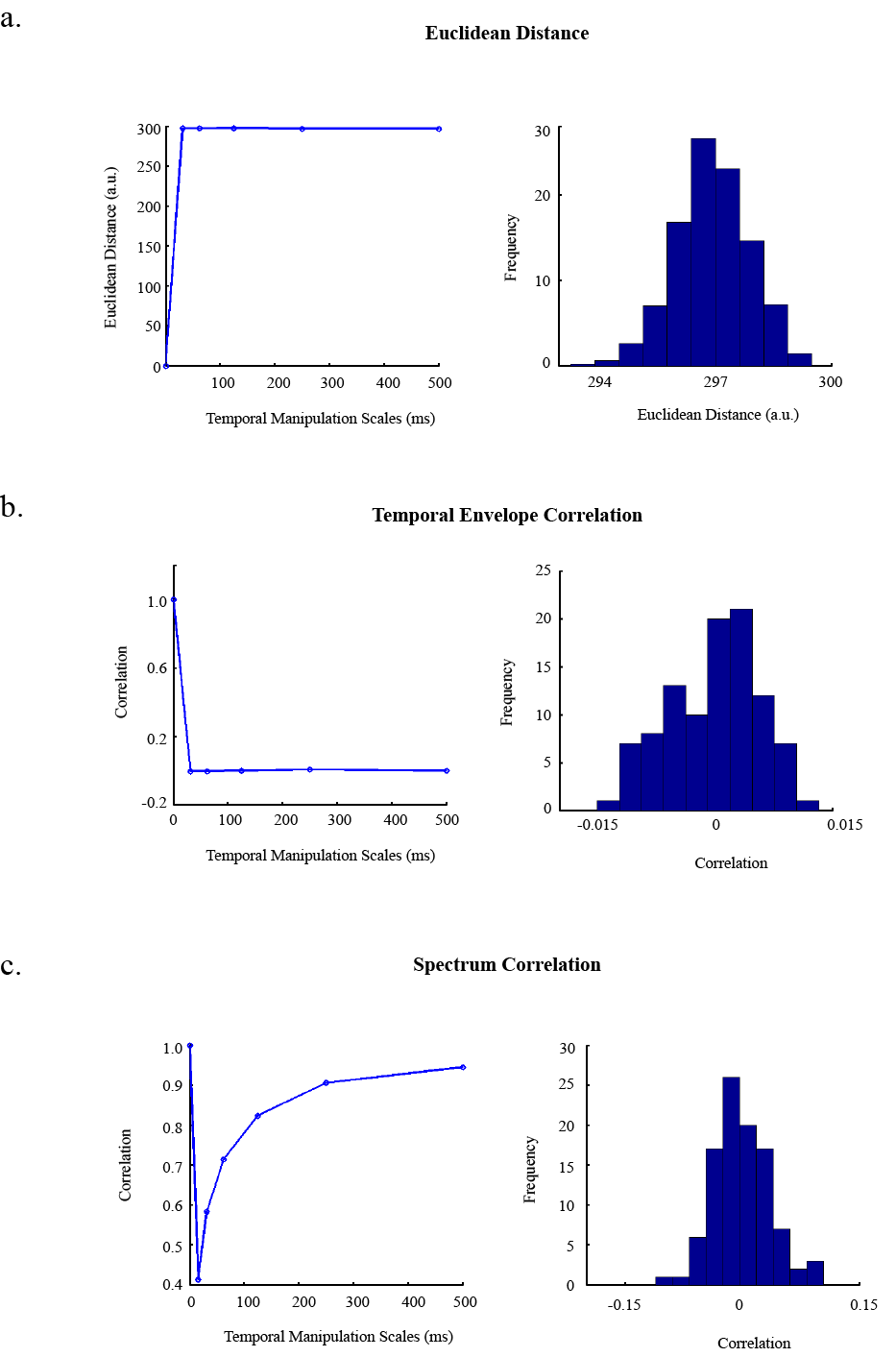

Supplement: FIGURE S1 — Physical similarity. (a) Left: the Euclidean distances between a noise segment in length of 0.5 s and its local temporal reversing versions at different temporal manipulation scales. Right: the distribution of Euclidean distance between two randomly generated noise segments, calculated from 500 different noise pairs. (b) Left: the correlations of the envelope of a noise segment in length of 0.5 s and the envelopes of its local temporal reversing versions at different temporal manipulation scales. Right: the distribution of the correlation between the envelopes of two randomly generated noise segments, calculated from 500 different noise pairs. (c) Left: the correlations of the spectrum equivalent bands pattern (ERB pattern) of a noise segment in length of 0.5 s and the envelopes of its local temporal reversing versions at different temporal manipulation scales. Right: the distribution of correlation between the ERB patterns of two randomly generated noise segments, calculated from 500 different noise pairs. [file Image_1.TIF]

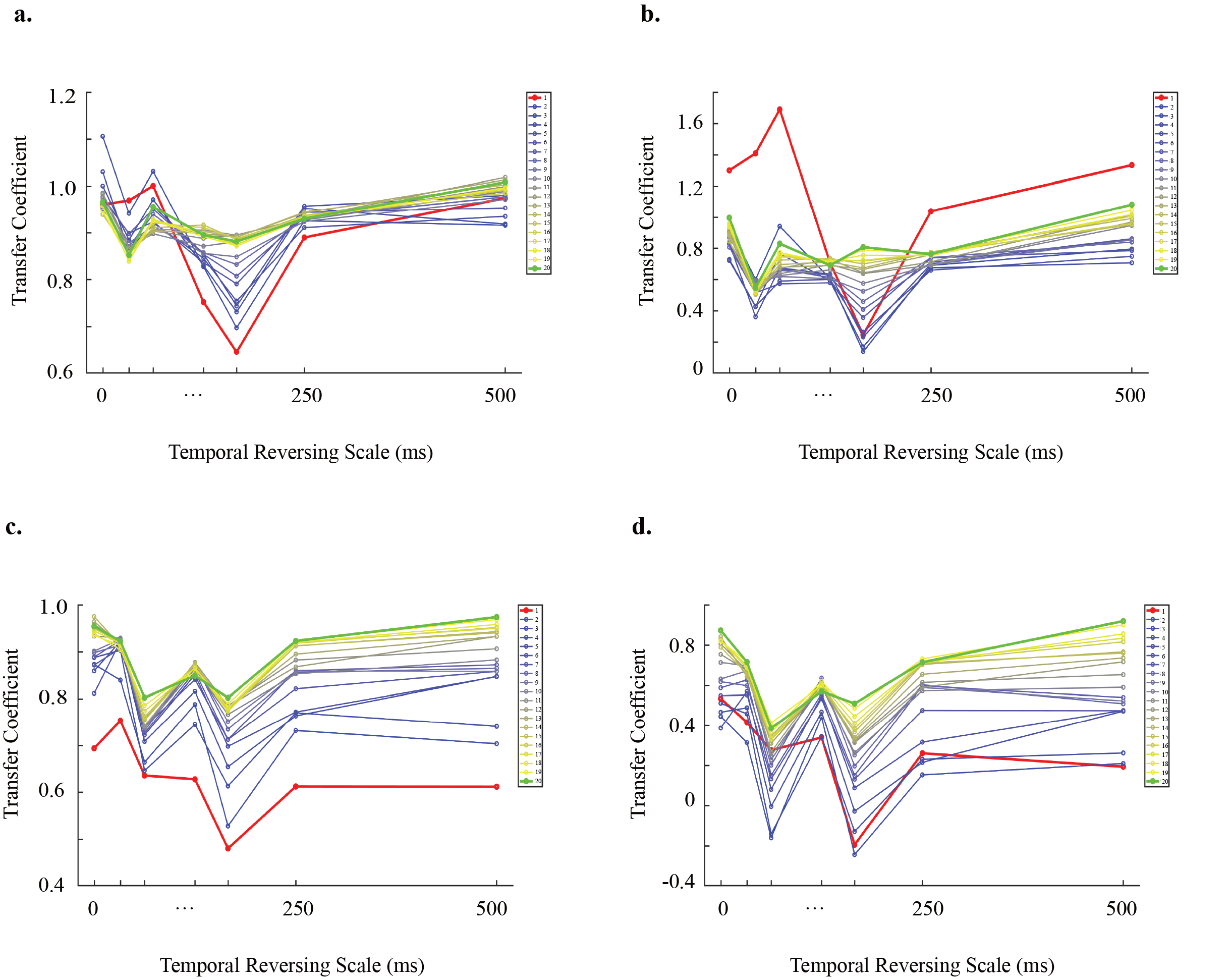

Supplement: FIGURE S2 — Memory-transferring coefficients calculated using other behavioral parameters. (a) AA-A′A′ memory-transferring coefficient as a function of temporal reversed scale, calculated using the hit rate averaged across different number of trials. (b) AA-A′A′ memory-transferring coefficient as a function of temporal reversed scale, calculated using d′, averaged across different number of trials. (c) AA-BB memory-transferring coefficient as a function of temporal reversed scale, calculated using the hit rate averaged across different number of trials. (d) AA-BB memory-transferring coefficient as a function of temporal reversed scale, calculated using d′, averaged across different number of trials. [file Image_2.TIF]
